# Characterization of Cornulin as a Molecular Biomarker for the Progression of Oral Squamous Cell Carcinoma

**DOI:** 10.7759/cureus.32210

**Published:** 2022-12-05

**Authors:** Mourad Kerdjoudj, Hilal Arnouk

**Affiliations:** 1 Medicine, Chicago College of Osteopathic Medicine, Midwestern University, Downers Grove, USA; 2 Pathology, Chicago College of Osteopathic Medicine, Midwestern University, Downers Grove, USA; 3 Pathology, College of Graduate Studies, Midwestern University, Downers Grove, USA; 4 Pathology, College of Dental Medicine-Illinois, Midwestern University, Downers Grove, USA; 5 Pathology, Chicago College of Optometry, Midwestern University, Downers Grove, USA; 6 Molecular Pathology, Precision Medicine Program, Midwestern University, Downers Grove, USA

**Keywords:** dysplasia, cornulin, cancer metastasis, tumor progression, prognostic biomarker, molecular biomarker, oral squamous cell carcinoma, head and neck cancer

## Abstract

Introduction

It has been shown that the expression of the epidermal differentiation marker, Cornulin, declines with the progression of squamous cell carcinomas of several tissue types.

Objectives

This study aims to examine Cornulin expression at the cellular level in various cell lines representative of the successive progression steps of oral squamous cell carcinoma (OSCC), a major type of head and neck cancer. This can pave the way for the utilization of this novel biomarker as a diagnostic and prognostic indicator for oral cancer and help guide treatment options.

Study design

Western blotting was performed to measure Cornulin expression levels using standardized cell lysates from four different cell lines representing the successive steps of OSCC progression. Specifically, primary gingival keratinocytes, dysplastic oral keratinocytes (DOK), squamous cell carcinoma 25 (SCC25) cells, and Detroit 562 cells were used to represent normal oral keratinocytes, DOKs, locally invasive OSCC cells, and metastatic OSCC cells, respectively.

Results

Cornulin expression was found to be downregulated with the progression from normal to premalignant to malignant cells. Quantitative analysis revealed that Cornulin is significantly downregulated by 3.4 folds in DOK cells, by 23.7 folds in SCC25 cells, and by 5.2 folds in Detroit 562 cells compared to normal gingival keratinocytes. Interestingly, Cornulin was upregulated by 4.5 folds in the metastatic Detroit 562 cell line compared to the locally invasive SCC25 cells.

Conclusion

Altogether, Cornulin proved to be differentially expressed at the cellular level in cell lines representative of the successive steps of OSCC progression. Specifically, we documented a gradual decrease in Cornulin expression with the progression from normal to premalignant to malignant cells. Notably, there is a significant increase in the expression of Cornulin in the metastatic cell line Detroit 562 compared to the locally invasive cell line SCC25, suggesting a possible correlation with the biological behavior and unique characteristics of these two different phenotypes.

## Introduction

Oral cancer consists of a family of neoplasms that could arise in any part of the oral cavity, pharyngeal region, or salivary glands [1]. Oral squamous cell carcinoma (OSCC) is the most common of oral cancers [2], making up more than an estimated 90% of all oral cancers [1]. The development of this carcinoma occurs via multiple steps involving the individual’s genetic predisposition in addition to environmental factors ranging from alcohol and tobacco use to viral infections and chronic inflammation [3]. Importantly, the morbidity and mortality rates of OSCC have not seen significant improvement over the last 30 years, with affected populations exhibiting a five-year survival rate of around 50% [3]. While the prognosis is best when diagnosis of OSCC is made early, most tend to be diagnosed in later stages of cancer, where the five-year survival rate does not exceed 12% [1]. Thus, early detection is the key to the effective treatment of OSCC. As histological morphology alone has been the primary method of diagnosing and predicting the prognosis and determining treatment [4], there is a level of subjectivity, and there is no single pathological feature that facilitates the detection of lesions with aggressive potential. This highlights the need for a novel method for diagnosing and monitoring the progression of OSCC lesions.

Cornulin, encoded by the *CRNN* gene, is a 495 amino acid protein belonging to the family of S100 fused-type proteins, is located on chromosome 1q21 locus, and has tumor suppressor characteristics [5]. It has previously been established that its expression declines in cervical [6], esophageal [7], cutaneous [8], and OSCCs [9,10]. Specifically, studies have demonstrated that Cornulin has markedly significant downregulation in tissue biopsies from oral cancer patients compared to the normal oral mucosa [11]. This study aimed to investigate the expression of Cornulin at the cellular level in an in vitro model of cell lines representing the stepwise progression of OSCC from the normal oral mucosa to the premalignant lesion, known as leukoplakia, to the locally invasive and metastatic phenotypes of OSCC. Here we have documented, for the first time, a gradual decrease in Cornulin expression in correlation to the stepwise progression from normal to premalignant to malignant keratinocytes of the oral cavity.

## Materials and methods

Cell culture

A total of four different cell lines were used to model the successive steps of OSCC progression. The squamous cell carcinoma 25 (SCC25) human OSCC cell line, Detroit 562 metastatic pharyngeal squamous cell carcinoma (SCC), and primary gingival epithelial cells were acquired from American Type Culture Collection (ATCC) (Manassas, VA), and the dysplastic oral keratinocytes (DOKs) were acquired from the European Collection of Authenticated Cell Cultures (ECACC) (Salisbury, United Kingdom) via Millipore Sigma (Darmstadt, Germany). All cell lines were cultured and maintained in their respective specialized media according to vendors’ instructions.

Western blot

The four cell lines were separately harvested for their total protein content and the protein concentration was determined using the BCA protein assay (Pierce Biotechnology, Rockford, IL). The cell lysate was prepared for the SDS-PAGE electrophoresis in radioimmunoprecipitation assay buffer (RIPA buffer) containing dithiothreitol (DTT), and the samples were then boiled for 5 minutes. An XCell SureLock system and Novex precast protein gels (Invitrogen, Carlsbad, CA) were used for the SDS-PAGE electrophoresis, and the gel was run at two different settings: the first at 60 V for 40 minutes and the second at 140 V for 1 hour and 35 minutes. Once complete, the wet transfer was set up using a Mini-PROTEAN Tetra system (Bio-Rad, Hercules, CA) and Immobilon-P PVDF transfer membranes (Millipore Sigma, Darmstadt, Germany). This was carried out at 100 V for 1 hour in a pre-chilled transfer buffer.

The resulting membranes were then blocked using 5% milk dissolved in 1x tris-buffered saline with 0.01% Tween (TBST). Membranes were blocked for 2 hours. The membranes were then incubated with the primary antibody in blocking buffer overnight at 4°C. A 1:100 dilution was used for the anti-Cornulin mouse monoclonal antibody (Catalog number: SC-514602, Santa Cruz Biotechnology, Santa Cruz, CA). Membranes were then washed and incubated with the secondary antibodies for 1 hour at room temperature. A 1:5,000 dilution was used for the mouse IgGκ light chain binding protein secondary antibody (Catalog number: SC-516102, Santa Cruz Biotechnology). Subsequent labeling of glyceraldehyde 3-phosphate dehydrogenase (GAPDH) was utilized as a loading control. A 1:10,000 dilution was used for GAPDH mouse monoclonal antibody (Catalog number 60004-1-Ig, Proteintech, Rosemont, IL). Membranes were subsequently imaged using a BioRad ChemiDoc XRS+ system (Bio-Rad) and Amersham ECL western blotting detection reagent (Little Chalfont, United Kingdom).

Quantitative analysis

The images produced by the BioRad ChemiDoc system (Bio-Rad) were analyzed using ImageJ software (National Institutes of Health, Bethesda, MD). The pixel intensity of each sample was assessed compared to the GAPDH control and plotted as a fold difference in protein expression of Cornulin compared to GAPDH. Sample sets of three repeats were compared to each other using an unpaired *t*-test.

## Results

**Figure 1:**
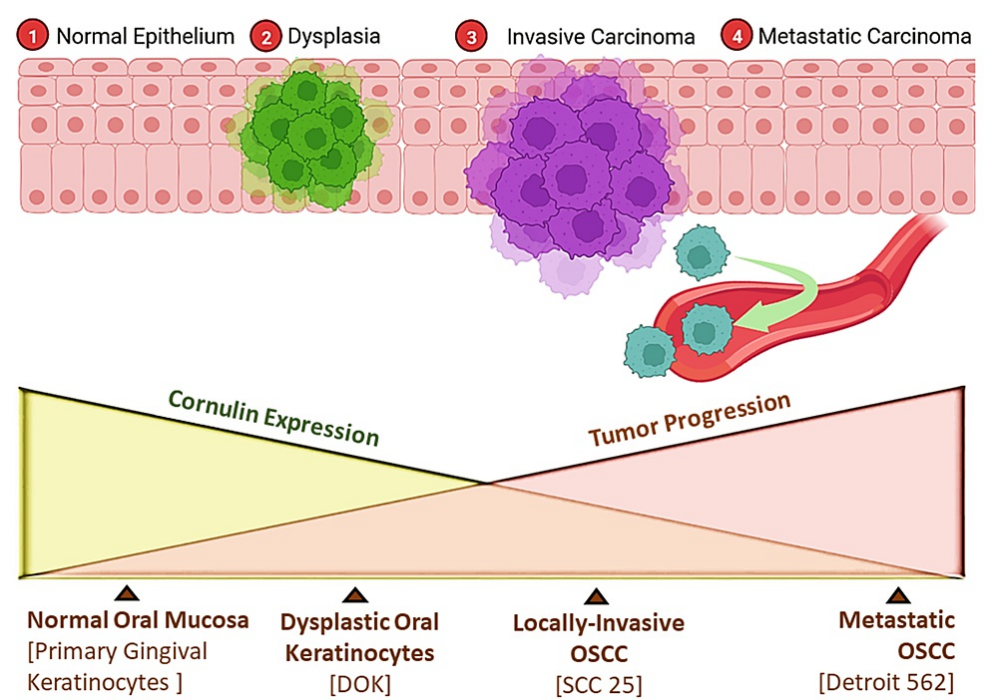
Expected trends in Cornulin expression with the progression of oral squamous cell carcinoma. Schematic graph showing the expected trends in Cornulin expression as dysplastic growth progresses toward malignant forms of oral squamous cell carcinoma. This original figure was drawn by authors using Microsoft Office PowerPoint and BioRender software.

Based on existing evidence on the role of Cornulin as a tumor suppressor gene product, we put forward a hypothesis predicting a gradual decrease in Cornulin expression in correlation to the stepwise progression from normal oral keratinocytes to premalignant keratinocytes to invasive and metastatic malignant OSCC keratinocytes (Figure 1).

To test our hypothesis, we utilized established human cell lines including primary gingival keratinocytes to model the normal oral mucosa, DOKs to represent the premalignant lesion known as leukoplakia, SCC25 cell line to represent locally invasive SCC cells, and Detroit 562 cell line as a representative for the metastatic phenotype (Table 1).

**Table 1:** Cell line descriptions, special characteristics, culture media, and sources.

Cell Line	Cell Line Description	Special Characteristics	Special Media	Source
Primary gingival keratinocytes	Female, 60 y/o	Normal	Dermal Cell Basal Medium, Keratinocyte Growth Kit	ATCC
Dysplastic oral keratinocyte	Mild/moderate dysplasia, male, 57 y/o	TP53 (mutant)	DMEM, FBS	Millipore Sigma/ECACC
SCC25	Oral squamous cell carcinoma, male, 70 y/o	CDKN2A, TP53 (mutant)	DMEM: F12, FBS	ATCC
Detroit 562	Metastatic oropharyngeal carcinoma, female, adult	CDKN2A, PIK3CA, TP53 (mutant)	EMEM, FBS	ATCC

**Figure 2:**
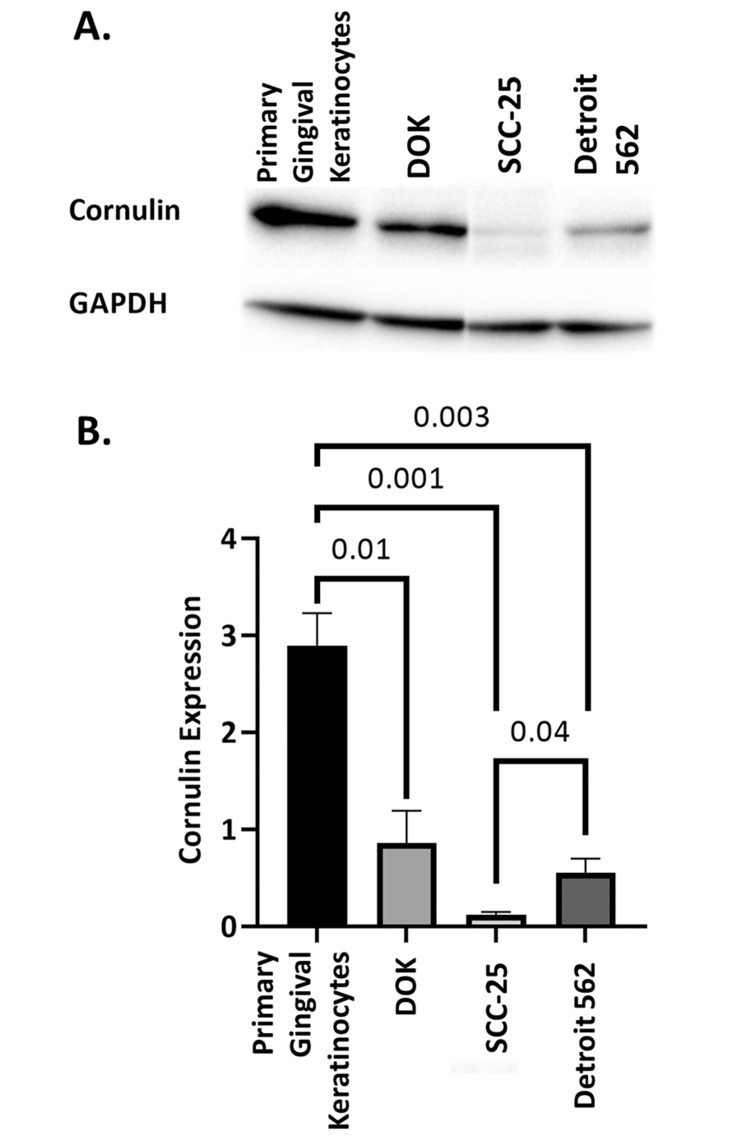
Quantitative analysis of Cornulin expression in the four cell lines representing the successive steps of oral squamous cell carcinoma progression. (A) Western blot showing expression of Cornulin and the GAPDH loading control in the four cell lines tested. (B) Comparison of Cornulin expression relative to GAPDH expression in normal oral keratinocytes (PGK), dysplastic oral keratinocytes (DOK), locally invasive oral squamous cell carcinoma cells (SCC25), and metastatic oral squamous cell carcinoma cells (Detroit 562). Error bars represent standard error of the mean (SEM), and values shown above brackets indicate significant p-values pertaining to the difference of expression between cell lines. GAPDH, glyceraldehyde 3-phosphate dehydrogenase

Western blot proteomic analysis on the four cell lines confirmed that Cornulin expression declines with the progression from normal oral keratinocytes to premalignant dysplastic keratinocytes to malignant OSCC cells (Figure 2A). Statistical analysis revealed that Cornulin was downregulated by 3.4 folds in the DOK cells when compared to the normal gingival keratinocytes (p = 0.01). Importantly, Cornulin expression was downregulated by 23.7 folds in the SCC25 cells compared to primary gingival keratinocytes (p = 0.001). Additionally, a comparison of normal gingival keratinocytes with metastatic squamous cell cancer cells represented by the Detroit 562 cell line shows that Cornulin expression was downregulated by 5.2 folds (p = 0.003). Interestingly, a significant increase of 4.5 folds was found in the metastatic Detroit 562 cell line compared to the locally invasive SCC25 cell line (p = 0.04) (Figure 2B).

## Discussion

Cornulin was first referred to as C1Orf10 and is known to be expressed in esophageal, oral, anal, cervical, and skin squamous epithelia [12,13]. It is also known as squamous epithelial heat shock protein 53 (SEP53) [14]. Cornulin binds calcium via its calcium-binding motif and is involved in the differentiation of keratinocytes in the late stages. Studies have demonstrated that Cornulin is downregulated in several types of SCCs including cervical [6], esophageal [7], cutaneous [8], and head and neck SCC [9,10]. It has also been shown that transfection of OSCC cell lines with Cornulin resulted in the reduction of proliferation of those cell lines with their arrest in the G1 phase of the cell [15] and that silencing Cornulin expression leads to improper G1 to S phase transition [5], thereby suggesting its role as a tumor suppressor gene due to its involvement in cell cycle regulation and prevention of tumorigenesis. Prior studies utilized methods such as immunohistochemistry (IHC), real-time reverse transcription polymerase chain reaction (RT-PCR), and western blotting to assess patterns of Cornulin expression between normal and cancerous tissue biopsies. Our study focused on quantitative analysis of Cornulin expression in established cell lines representing the successive steps of OSCC tumor progression, namely non-cancerous, dysplastic, locally invasive, and metastatic oral lesions. Thus, making the case for the potential utility of Cornulin as a diagnostic and prognostic indicator for the progression of OSCC.

Significant differences in Cornulin expression were observed in dysplastic keratinocytes (DOK), locally invasive cancerous cells (SCC25), and metastatic cancerous cells (Detroit 562) in comparison to the normal gingival keratinocytes. Interestingly, a statistically significant increase in Cornulin expression was noted between the locally invasive SCC25 cells and the metastatic Detroit 562 neoplastic cells, possibly pointing to a role for Cornulin in the process of metastasis or perhaps as a result of the natural selection of few cells with metastatic abilities and higher than average Cornulin levels from the heterogeneous cell population of the primary tumor with variable Cornulin expression levels.

A limitation of this study is the reliance on established human cell lines to represent the steps in progression of oral squamous carcinoma lesions. Although in vitro models for tumor progression, such as the immortalized, dysplastic, and cancer cell lines used in this study, have the advantages of fast growth rate [16], unlimited starting material, relative homogeneity, and reproducibility, they may not recapitulate the exact changes that occur in patients' tissues since isolated cells do not account for the effects of the tumor microenvironment and the interactions with stromal, endothelial, inflammatory cells, and normal surrounding tissues. Additionally, they do not model the survival pressure exerted by the tumor microenvironment and the selection process of the fittest sub-clones of the heterogeneous population of malignant cells, including cancer stem cells, within tumors. Therefore, in the next phase of our lab investigations, we aim to perform immunhistochemical analysis of Cornulin in patients' specimens of oral epithelial tissues, including normal, inflamed, dysplastic, and SCC of varying clinicopathological characteristics. Additionally, we plan on re-examining the Cornulin expression patterns after implanting these human tumor cell lines into immunocompromised mice.

In the clinical practice, OSCC, leukoplakia, and several non-cancerous oral lesions are difficult to distinguish from each other by physical examination alone [17,18]. This highlights the importance of identifying novel molecular biomarkers that can be used to improve the accuracy of diagnostic tests for oral neoplastic lesions. Moreover, oral cancer prognosis and outcome predictive parameters rely heavily on the TNM staging system that is based on local invasion, regional lymph node involvement, and distant metastases [19]. However, patients with similar TNM staging classification often have different clinical outcomes, indicating that reliance on TNM clinical staging alone might not be a sufficient measure for prognosis [20]. Thus, it will be highly desirable to characterize molecular biomarkers, such as Cornulin, that can predict clinical outcomes, monitor disease progression and relapse, and potentially help guide treatment options.

## Conclusions

This study has demonstrated significant quantitative differences in the expression of Cornulin across the successive steps of OSCC progression. These findings allude to the potential utility of Cornulin as a predictive and prognostic indicator. Follow-up studies to monitor Cornulin levels throughout the clinical course of disease and relative survival rate data are needed to firmly establish this promising biomarker in the clinical setting to supplement current pathological assessment and prognosis for patients afflicted with oral cancer.
